# Concise Synthesis of Catechin Metabolites 5-(3′,4′-Dihydroxyphenyl)-γ-valerolactones (DHPV) in Optically Pure Form and Their Stereochemical Effects on Skin Wrinkle-Reducing Activities

**DOI:** 10.3390/molecules25081970

**Published:** 2020-04-23

**Authors:** Joonseong Hur, A-Ram Kim, Hyun Su Kim, Changjin Lim, Taewoo Kim, Tae-Aug Kim, Jaehoon Sim, Young-Ger Suh

**Affiliations:** 1 Natural Products Research Institute, Korea Institute of Science and Technology (KIST), 679 Saimdang-ro, Gangneung 25451, Korea; hjs1120@kist.re.kr; 2 Department of Biochemistry, School of Medicine, CHA University, 335 Pangyo-ro, Bundang-gu, Seongnam 13496, Gyeonggi-do, Korea; tmfdkdjssl@hanmail.net (A.-R.K.); kimuara@msn.com (T.-A.K.); 3 Department of Pharmacy, College of Pharmacy and Institute of Pharmaceutical Sciences, CHA University, 120 Haeryong-ro, Pocheon 11160, Gyeonggi-do, Korea; khs8812@snu.ac.kr (H.S.K.); taewookim@snu.ac.kr (T.K.); 4 College of Pharmacy, Jeonbuk National University, Jeonju 54896, Korea; limcj@jbnu.ac.kr; 5 College of Pharmacy, Chungnam National University, Daejeon 34134, Korea

**Keywords:** catechin metabolite, γ-valerolactone, concise synthesis, skin wrinkle-reducing activity

## Abstract

A concise and scalable synthetic route for optically pure (4*S*) and (4*R*)-5-(3′,4′-dihydroxyphenyl)-γ-valerolactones (DHPVs), catechin metabolites, has been developed via the efficient construction of a γ-valerolactone moiety from hexenol. Noticeably, the different skin wrinkle-reducing activities of each metabolite were revealed via our unique syntheses of DHPVs in an enantiomerically pure form.

## 1. Introduction

**Figure 1:**
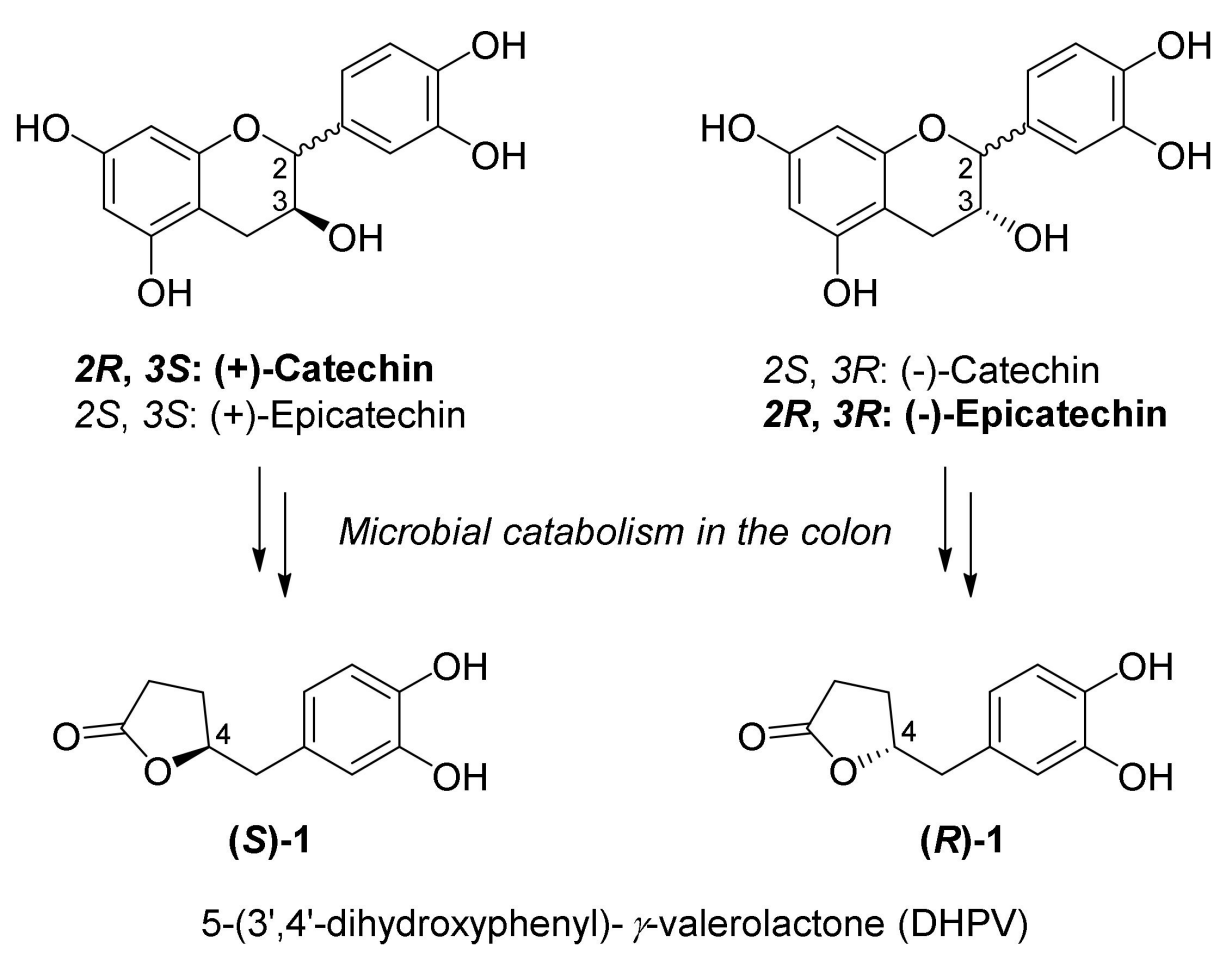
Structures of catechins and their metabolite DHPVs.

Flavan-3-ols are the most abundant class of naturally occurring polyphenols widely found in dietary plants, especially green tea, red wine, and cocoa extract [1]. The monomeric flavan-3-ols structurally possess a 2-phenyl-3,4-dihydro-2*H*-chromen-3-ol skeleton with two chiral centers at the C2 and C3 position (Figure 1), and each natural product including (*epi*)afzelechin, (*epi*)catechin, and (*epi*)gallocatechin contains a different number of hydroxyl groups on the aromatic ring. This class of compounds has received significant attention for their protective effects against various chronic diseases, such as cancer, cardiovascular disease, diabetes, and neurodegenerative disease [2]. However, after consumption of polyphenol-rich foods, only a few flavan-3-ols are able to directly reach the circulatory system due to their poor absorbability [3,4]. Instead, substantial quantities of intact flavan-3-ols are degraded into simple metabolites by the microbiota in the colon and then absorbed into the blood stream [5]. Therefore, synthetic and biological studies focused on each single metabolite of the flavan-3-ols are essential for understanding the beneficial effects of polyphenols on human health.

5-(3′,4′-Dihydroxyphenyl)-*γ*-valerolactone (DHPV; **1**) is a major metabolite of (*epi*)catechin generated by microbial degradation in the colon (Figure 1) [6,7]. The C4-stereochemistry of each enantiomer is derived from the C3 configuration of parent flavan-3-ols and both enantiomers are naturally found. Upon incubation of naturally abundant (+)-catechin and (−)-epicatechin with rat intestinal microbiota, the C3 configuration of each flavan-3-ol was retained during the catabolism (Figure 1) [8]. Since the first report on the metabolic fate of (+)-catechin in rabbits by Watanabe et al. [9], there have been intensive pharmacokinetic studies on DHPV [10,11,12] after the intake of a single flavan-3-ol or polyphenol-rich foods in various living organisms for the past half century. However, investigation on its biological effects were relatively rare, except for a few reports [13,14,15,16]. Despite the lack of studies on DHPVs’ role in the biological system, several papers recently reported that DHPV exhibits a protective effect against UV-induced skin inflammation [17] or photoaging [18]. Particularly, Lee and Kim et al. [18] recently reported that oral administration of DHPV reduced UV-induced skin-wrinkling via the regulation of genes involved in dermal matrix maintenance. These observations encouraged us to develop a novel protocol for the synthesis of DHPV to secure optically pure metabolites in sufficient quantities to utilize their potential as promising cosmeceutical ingredients. 

Herein, we report a highly concise and scalable synthetic route for optically pure DHPVs. Moreover, inspired by evidence that the bioavailability of (*epi*)catechin depends on their stereochemistry [19], we envisioned that (*R*)- and (*S*)-DHPV might exhibit different *anti*-photoaging activities. Thus, we describe our unique synthesis of both DHPV enantiomers and their MMP-1 [20,21] inhibitory activities, which is a representative biomarker of UV-induced skin wrinkling [22].

## 2. Results and Discussion

**Figure 2:**
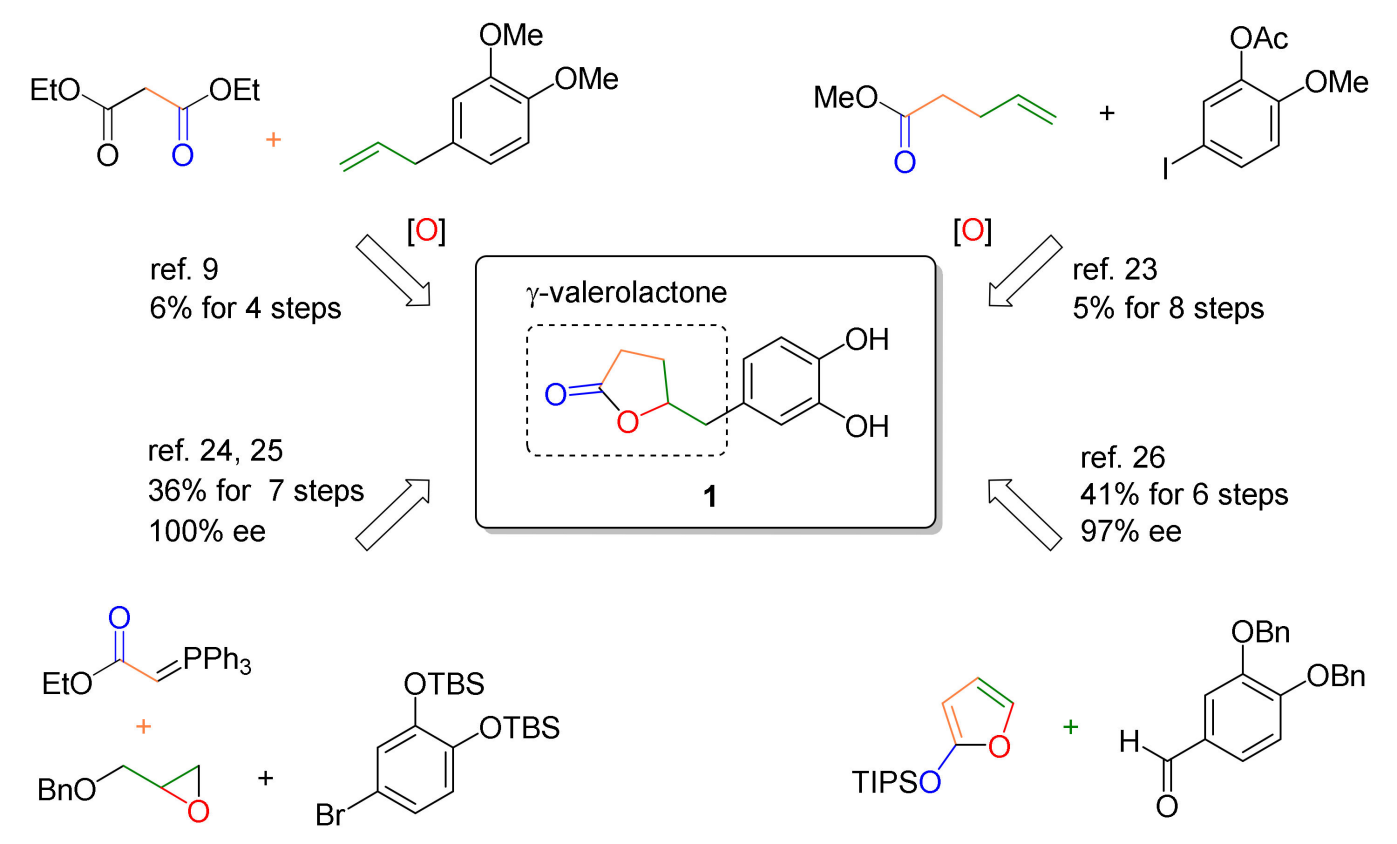
Previous synthetic approaches toward DHPV.

The syntheses of racemic or optically active DHPV were reported by several groups for further biological investigation or testing of their own catalytic system, as summarized in Figure 2. In 1959, Watanabe et al. achieved the first total synthesis of racemic DHPV in 6% overall yield over four steps [9]. The key step included an epoxide opening/lactonization/decarboxylation cascade under vigorous refluxing conditions to obtain the γ-valerolactone core. Lambert et al. reported another synthesis of racemic DHPV to secure the tea catechin metabolites for testing their anticancer and anti-inflammatory activities [23]. The γ-valerolactone core was constructed in a stepwise manner, including epoxidation, regioselective epoxide opening, hydrolysis, and dehydrative lactonization, which result in low overall yield. The first synthesis of optically pure DHPVs was accomplished by Nakajima et al. in 2008 [24] and improved in 2010 [25], using (*R*)- and (*S*)-benzyl glycidyl ether as chiral sources. Although the γ-valerolactone formation still included protection/deprotection and homologation steps, overall yield was substantially increased up to 36%. Most recently, Curti et al. reported an enantioselective method [26]. Their elegant catalytic approach exploiting vinylogous Mukaiyama aldol reaction was able to introduce γ-lactone moiety onto benzylic position in a single step with moderate to high stereoselectivity, although global steps was not successfully reduced.

Collectively, these synthetic routes feature the addition of requisite carbons onto the aromatic part, followed by the construction of the γ-valerolactone structure. However, despite excellent previous syntheses, the desire for efficient DHPV preparation in terms of number of steps, overall yield, scalability, mild conditions, and stereocontrol still remains. In particular, the previous methods were hampered by multiple steps or harsh conditions to construct the γ-valerolactone core.

To overcome these drawbacks, we employed an oxidative lactonization [27] of a hexenol *rac*-**4** generated by a regioselective epoxide opening reaction of *rac*-**3** [24,25,28], which is able to conveniently provide both enantiomers of DHPV with excellent enantiomeric excess and in a practical and efficient manner.

**Scheme 1:**
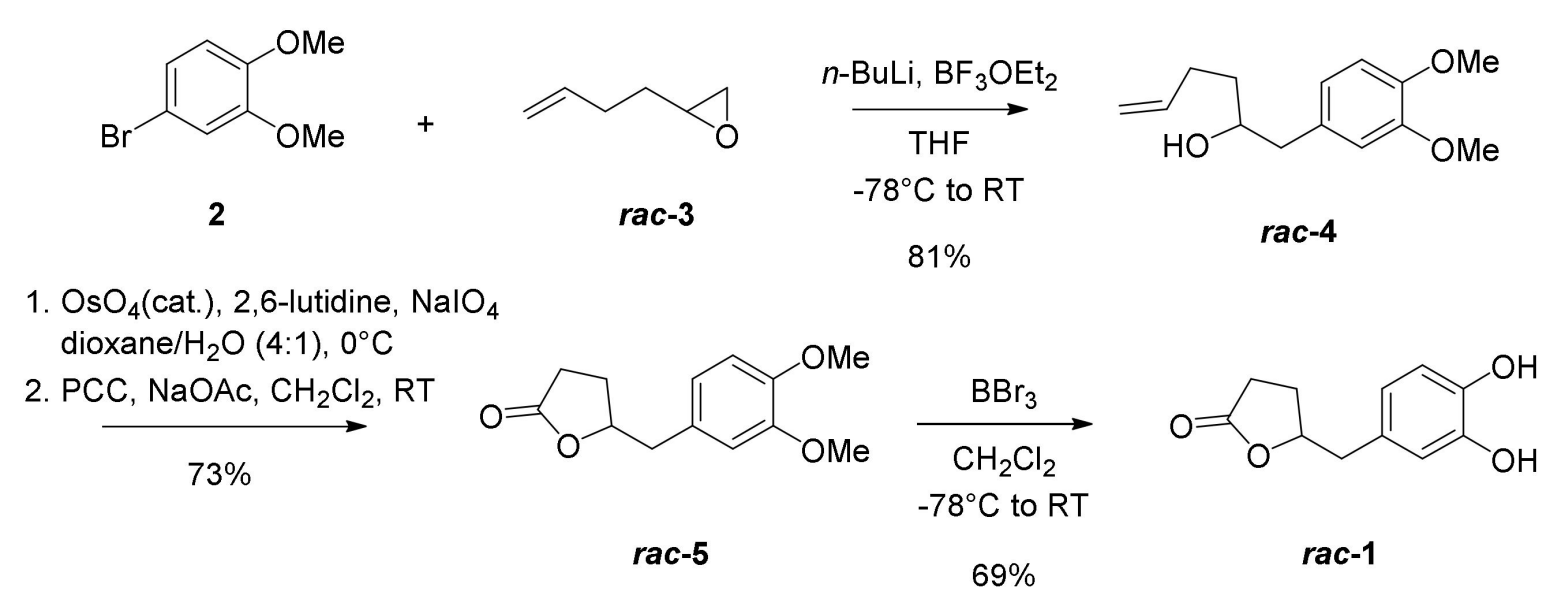
Synthesis of racemic DHPV (*rac*-**1**).

We initially examined our synthetic strategy by synthesizing racemic DHPV (Scheme 1). Aryl bromide **2**, which is commercially available, was lithiated and coupled with racemic epoxide **3** to afford hexenol **4** in 81% yield with perfect regioselectivity. To obtain the γ-valerolactone core in a concise way, we investigated several conditions of oxidative lactonization of **4**, as summarized in Table 1. A classical three-step protocol successfully afforded the desired lactone **5** via a sequence of dihydroxylation, oxidative cleavage, and corresponding lactol oxidation (71%; entry 1). Confirming synthetic efficiency and practicality, two-step protocols were examined by combining dihydroxylation-oxidative cleavage sequence in a single operation (entry 2–4). Although the yield was slightly decreased, the use of NaIO_4_ and a catalytic amount of OsO_4_ gave moderate yield of desired product **5** (51%, entry 2). As expected, the addition of base additives [29], such as pyridine or 2,6-luthidine, improved yield up to 73% (entry 3 and 4). Hexenol **4** did not undergo the one-step oxidative lactonization using catalytic OsO_4_ and Oxone^®^ as a co-oxidant [27].

With optimized condition in hand for the synthesis of lactone **5** (entry 4), demethylation of **5** using boron tribromide was successfully conducted to provide 1.2 g of the desired phenolic metabolite **1** in 69% yield.

**Scheme 2:**
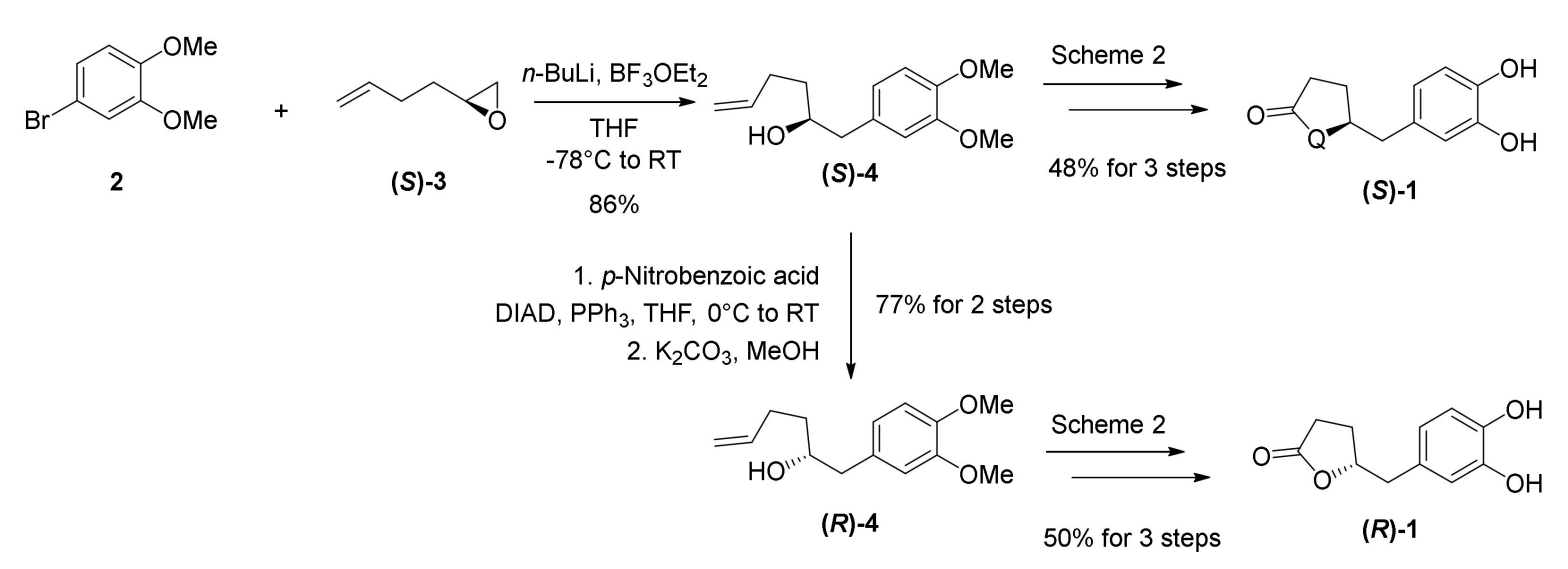
Synthesis of optically pure DHPVs ((*S*)-**1** and (*R*)-**1**).

We next turned our attention to the syntheses of optically pure DHPVs (Scheme 2). Optically active (*S*)-**1** was conveniently obtained from optically pure epoxide (*S*)-**3** via our established synthetic route. For the synthesis of (*R*)-**1**, as an antipode of (*S*)-**1**, we initially inversed the stereochemistry of the C4-stereocenter in the secondary alcohol (*S*)-**4** by a Mitsunobu reaction, followed by methanolysis to yield alcohol (*R*)-**4**. We obtained (*R*)-**1** via the same procedure applied for the synthesis of (*S*)-**4**. NMR spectra (800MHz for ^1^H-NMR) and chiral HPLC analysis of the synthetic DHPVs (Supporting Information, Figures S5–S7) confirmed almost perfect purity for biological experiments including skin-protection against UV-irradiation. 

We finally examined the stereochemical effects on the skin wrinkle-reducing activity of the DHPVs, which was previously reported by us [18]. The ROS in skin tissue is frequently increased by UV radiation, which consequently causes an increase in expression of MMP-1 and transcription activity of AP-1. AP-1 is a transcription factor of MMP-1, which breaks down collagen fiber, and is a composition of skin dermis extracellular matrix. The degradation of collagen causes structural collapse of skin dermis. Therefore, the increase of AP-1 and MMP-1 induces skin wrinkles as a phenotype of photo-aging. For these reasons, we envisioned that MMP-1 could be an appropriate biomarker to evaluate the skin-protecting activities of the synthetic DHPVs from UV-irradiation. 

**Figure 3:**
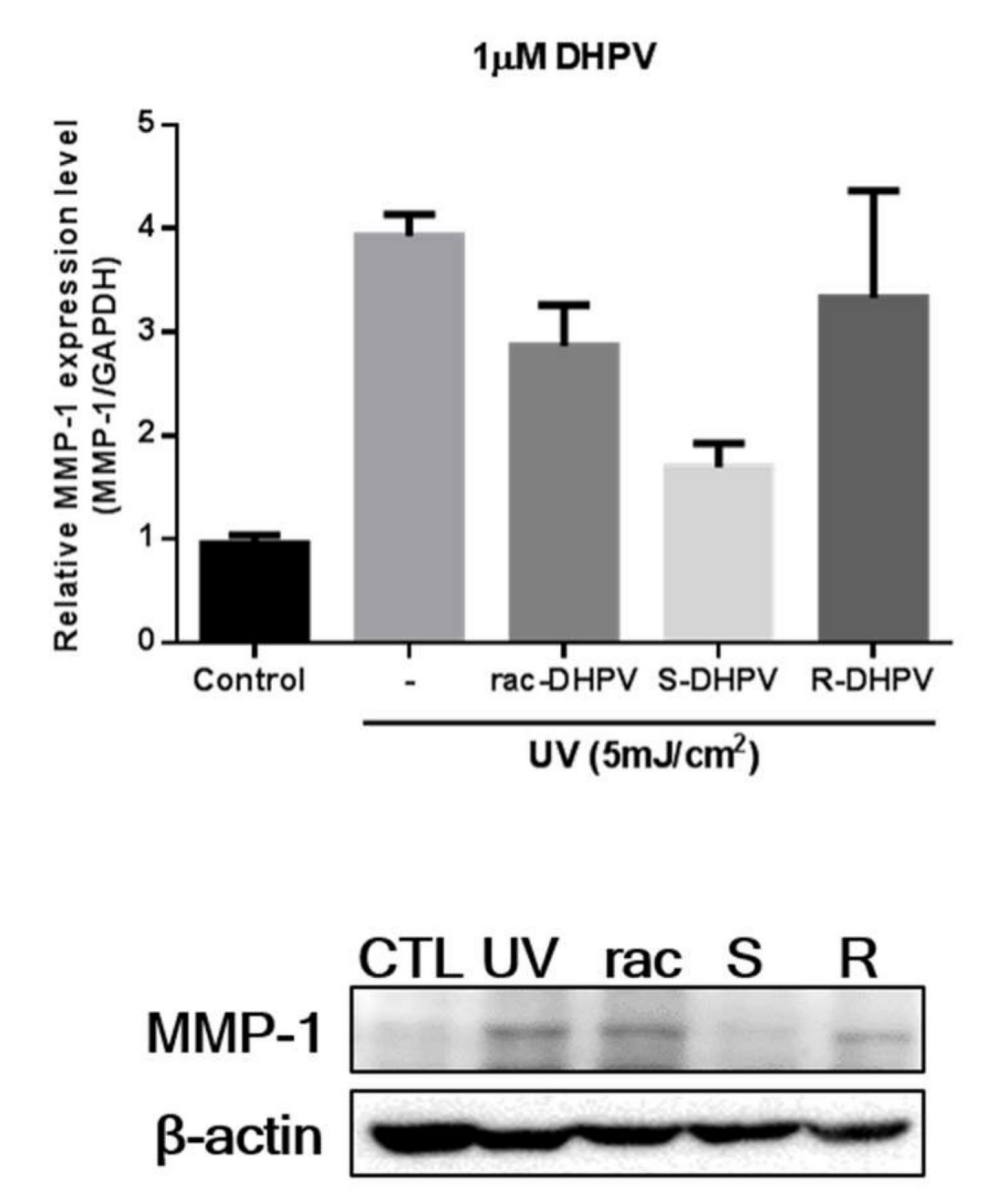
Suppressive activities of the racemic and optically pure DHPVs against UV-induced MMP-1 expression assessed by quantitative real-time PCR (**top**) and western blot analysis (**bottom**).

After the human dermal fibroblasts (HDF) was incubated with the tested metabolites at 1 μM concentration under UV light for a day, the suppressive activities of the racemic, (*S*), and (*R*)-DHPVs (**1**) against the photoinduced MMP-1 protein expression [22] was evaluated. Interestingly, the (*S*)-DHPV isomer revealed inhibitory effects two-folds greater than the (*R*)-DHPV isomer in quantitative real-time PCR and western blot assay, as summarized in Figure 3. Currently, we do not fully understand the stereochemical effects of DHPVs on their suppressive activities against UV-induced MMP-1 expression. However, these results confirmed that the metabolites of the flavan-3-ols possess different suppressive effects in the photoinduced MMP-1 protein expression, which are quite dependent on the C4-stereocenter of the DHPV.

## 3. Materials and Methods

### 3.1. General Experiment

Unless noted otherwise, all starting materials, reagents, and solvents were obtained from commercial suppliers and used without further purification. (*S*)-(−)-1,2-Epoxy-5-hexene was purchased from BOC Sciences. Flash column chromatography was performed using silica gel 60 (230–400 mesh, Merck, Kenilworth, NJ, USA) with the indicated solvents. Thin-layer chromatography was performed using 0.25 mm silica gel plates (Merck, Kenilworth, NJ, USA). Optical rotations were measured with JASCO P-2000 digital polarimeter (JASCO, Easton, MD, USA) at ambient temperature using 10 mm cell of 0.1 mL capacity. Infrared spectra were recorded on a JASCO FT-IR-4200 spectrometer (JASCO, Easton, MD, USA). High resolution mass spectra were obtained with Agilent Q TOF 6530 (Agilent, Santa Clara, CA, USA). High performance liquid chromatography (HPLC) experiments were performed with Waters 1525 binary pump (Waters, Milford, MA, USA) and Waters 2489 UV/Visible detector (Waters, Milford, MA, USA) at 35 °C. The ^1^H- and ^13^C-NMR spectra were recorded using BRUKER AVANCE-800 (Bruker, Billerica, MA, USA). Chemical shifts are expressed in parts per million (ppm, δ) downfield from tetramethylsilane and are referenced to the deuterated solvent (CHCl_3_). ^1^H-NMR data were reported in the order of chemical shift, multiplicity (s, singlet; d, doublet; t, triplet; q, quartet; ABq, AB quartet; quint, quintet; m, multiplet and/or multiple resonances), number of protons, and coupling constant in hertz (Hz).

### 3.2. Experimental Part

#### 3.2.1. Preparation of 1-(3,4-dimethoxyphenyl)hex-5-en-2-ol (rac-**4**)

To a solution of 4-bromo-1,2-dimethoxybenzene (3.0 g, 13.8 mmol) in THF (15 mL) was added *n*-butyllithium (2.5 M in hexane, 6.1 mL, 15.2 mmol) dropwise at −78 °C. The mixture was stirred for 40 min at the same temperature and BF_3_OEt_2_ (1.9 mL, 15.2 mmol) and 2-(but-3-en-1-yl)oxirane (1.4 g, 13.8 mmol) was consecutively added. After stirring for 24 h at room temperature, the reaction mixture was quenched with water and extracted with EtOAc. The combined organic layer was washed with aqueous NaHCO_3_, dried over MgSO_4_, and concentrated *in vacuo*. The residue was purified by flash column chromatography (EtOAc/Hexane = 1:5) to give 2.6 g (81%) of *rac*-**4** as a colorless oil.

^1^H-NMR (800 MHz, CDCl_3_) δ 6.80 (d, *J* = 8.1 Hz, 1H), 6.73 (dd, *J* = 8.1, 1.9 Hz, 1H), 6.71 (d, *J* = 1.9 Hz, 1H), 5.82 (ddt, *J* = 16.9, 10.2, 6.7 Hz, 1H), 5.03 (ddd, *J* = 17.1, 3.4, 1.6 Hz, 1H), 4.96 (ddd, *J* = 10.3, 2.9, 1.2 Hz, 1H), 3.85 (s, 3H), 3.84 (s, 3H), 3.79 (ddt, *J* = 12.5, 8.4, 4.3 Hz, 1H), 2.76 (dd, *J* = 13.7, 4.2 Hz, 1H), 2.57 (dd, *J* = 13.7, 8.6 Hz, 1H), 2.28 – 2.21 (m, 1H), 2.15 (ddt, *J* = 6.8, 5.6, 4.0 Hz, 1H), 1.66 – 1.53 (m, 4H).; ^13^C-NMR (200 MHz, CDCl_3_) δ 148.9, 147.7, 138.4, 130.9, 121.3, 114.8, 112.5, 111.3, 72.1, 55.9, 55.8, 43.6, 35.8, 30.1.; HRMS (FAB+) calcd for C_14_H_20_O_3_ (M^+^) 236.1412, found 236.1417; IR (thin film, neat) *ν*_max_ 3493, 2935, 1517, 1262, 1236, 1156, 1141, 1029 cm^−1^.

#### 3.2.2. Preparation of 5-(3,4-dimethoxybenzyl)dihydrofuran-2(3H)-one (rac-**5**)

To a solution of 1-(3,4-dimethoxyphenyl)hex-5-en-2-ol (2.6 g, 10.9 mmol) in 1,4-dioxane (10 mL) and water (3 mL) was added 2,6-lutidine (2.6 mL, 21.8 mmol), OsO_4_ (0.1 M in toluene, 1.1 mL, 0.1 mmol), and NaIO_4_ (9.4 g, 43.7 mmol) at 0 °C. After stirring for 17 h at room temperature, the reaction mixture was quenched with water and extracted with CH_2_Cl_2_. The combined organic layer was washed with brine, dried over MgSO_4_, and concentrated *in vacuo* to afford the corresponding lactol. The lactol was used in the next step without further purification.

To a solution of the resulting lactol in CH_2_Cl_2_ (13 mL) was added NaOAc (1.8 g, 21.8 mmol) and PCC (4.7 g, 21.8 mmol) at room temperature. After stirring for 1 h at the same temperature, the reaction mixture was concentrated and the residue was directly purified by flash column chromatography (EtOAc/Hexane = 1:2) to give 1.9 g (73%) of *rac*-**5** as white solid.^1^H-NMR (800 MHz, CDCl_3_) δ 6.78 (d, *J* = 8.0 Hz, 1H), 6.73 (dd, *J* = 8.0, 2.0 Hz, 1H), 6.72 (d, *J* = 1.9 Hz, 1H), 4.72–4.67 (m, 1H), 3.84 (s, 3H), 3.83 (s, 3H), 2.94 (dd, *J* = 14.2, 5.9 Hz, 1H), 2.87 (dd, *J* = 14.2, 6.0 Hz, 1H), 2.41 (ddd, *J* = 17.8, 9.7, 8.9 Hz, 1H), 2.29 (ddd, *J* = 17.7, 9.5, 4.8 Hz, 1H), 2.22 (dddd, *J* = 12.8, 9.8, 6.9, 4.8 Hz, 1H), 1.91 (dtd, *J* = 12.9, 9.2, 7.3 Hz, 1H).; ^13^C-NMR (200 MHz, CDCl_3_) δ 177.0, 148.9, 148.0, 128.3, 121.5, 112.6, 111.2, 80.8, 55.8, 55.8, 40.7, 28.6, 26.8.; HRMS (FAB+) calcd for C_13_H_16_O_4_ (M^+^) 236.1049, found 236.1045; IR (thin film, neat) *ν*_max_ 2938, 1770, 1517, 1465, 1262, 1238, 1178, 1159, 1144, 1027 cm^−1^.

#### 3.2.3. Preparation of 5-(3,4-dihydroxybenzyl)dihydrofuran-2(3H)-one (rac-**1**)

To a solution of 5-(3,4-dimethoxybenzyl)dihydrofuran-2(3*H*)-one (1.9 g, 8.0 mmol) in CH_2_Cl_2_ (8 mL) was added BBr_3_ (1.0 M in CH_2_Cl_2_, 33.6 mL, 33.6 mmol) at −78 °C. After stirring for 10 h at room temperature, the reaction mixture was quenched with water and extracted with EtOAc. The combined organic layer was washed with brine, dried over MgSO_4_, and concentrated *in vacuo*. The residue was purified by flash column chromatography (EtOAc/Hexane = 2:1) to give 1.2 g (69%) of *rac*-**1** as white solid. ^1^H-NMR (800 MHz, MeOD) δ 6.69 (d, *J* = 8.0 Hz, 1H), 6.68 (d, *J* = 2.0 Hz, 1H), 6.55 (dd, *J* = 8.0, 2.1 Hz, 1H), 4.75–4.69 (m, 1H), 2.86 (dd, *J* = 14.1, 6.1 Hz, 1H), 2.78 (dd, *J* = 14.1, 6.1 Hz, 1H), 2.50–2.44 (m, 1H), 2.32 (ddd, *J* = 17.8, 9.5, 4.8 Hz, 1H), 2.23 (dddd, *J* = 12.7, 9.8, 6.9, 4.8 Hz, 1H), 1.97–1.92 (m, 1H).; ^13^C-NMR (200 MHz, MeOD) *δ* 181.2, 147.1, 146.0, 129.9, 122.7, 118.5, 117.2, 84.1, 42.2, 30.3, 28.6.; HRMS (FAB+) calcd for C_11_H_13_O_4_ [M + H] ^+^ 209.0814, found 209.0820; IR (thin film, neat) *ν*_max_ 3377, 1753, 1520, 1446, 1285, 1230, 1191, 1116 cm^−1^.

#### 3.2.4. Preparation of (S)-1-(3,4-dimethoxyphenyl)hex-5-en-2-ol ((S)-**4**)

Alcohol (*S*)-**4** was prepared via the same procedure as that of *rac*-**4** using (*S*)-(−)-1,2-epoxy-5-hexene as a starting material. [α${]}_{D}^{20}$ 17.5 (*c* 0.15, CHCl_3_).

#### 3.2.5. Preparation of (R)-1-(3,4-dimethoxyphenyl)hex-5-en-2-ol ((R)-**4**)

To a solution of (*S*)-1-(3,4-dimethoxyphenyl)hex-5-en-2-ol (297 mg, 1.3 mmol) in THF (13 mL) was added *p*-nitrobenzoic acid (315 mL, 1.9 mmol), DIAD (0.4 mL, 1.9 mmol), and PPh_3_ (496 mg, 1.9 mmol) at 0 °C. After stirring overnight at room temperature, the reaction mixture was concentrated and the residue was directly purified by flash column chromatography (EtOAc/Hexane = 1:9) to give 496 mg (100%) of (*R*)-1-(3,4-dimethoxyphenyl)hex-5-en-2-yl-4-nitrobenzoate as a yellow oil.

To a solution of above (*R*)-nitrobenzoate (496 mg, 1.3 mmol) in MeOH (13 mL) was added K_2_CO_3_ (267 mg, 1.9 mmol) at room temperature. After stirring for 1 h at the same temperature, the reaction mixture was quenched with water and extracted with EtOAc. The combined organic layer was washed with brine, dried over MgSO_4_, and concentrated *in vacuo*. The residue was purified by flash column chromatography (EtOAc/Hexane = 1:5) to give 236 mg (77%) of (*S*)-**4** as a colorless oil. [α${]}_{D}^{20}$ −20.5 (*c* 1.0, CHCl_3_).

#### 3.2.6. Preparation of (S)-5-(3,4-dimethoxybenzyl)dihydrofuran-2(3H)-one ((S)-**5**)

Lactone (*S*)-**5** was prepared via the same procedure as that of *rac*-**5** using (*S*)-**4** as a starting material. [α${]}_{D}^{20}$ 21.4 (*c* 1.0, CHCl_3_).

#### 3.2.7. Preparation of (R)-5-(3,4-dimethoxybenzyl)dihydrofuran-2(3H)-one ((R)-**5**)

Lactone (*R*)-**5** was prepared via the same procedure as that of *rac*-**5** using (*R*)-**4** as a starting material. [α${]}_{D}^{20}$ −29.3 (*c* 1.0, CHCl_3_).

#### 3.2.8. Preparation of (S)-5-(3,4-dihydroxybenzyl)dihydrofuran-2(3H)-one ((S)-**1**)

DHPV (*S*)-**1** was prepared via the same procedure as that of *rac*-**1** using (*S*)-**5** as a starting material. [α${]}_{D}^{20}$ 8.1 (*c* 1.0, MeOH).

#### 3.2.9. Preparation of (R)-5-(3,4-dihydroxybenzyl)dihydrofuran-2(3H)-one ((R)-**1**)

DHPV (*R*)-**5** was prepared via the same procedure as that of *rac*-**1** using (*R*)-**5** as a starting material. [α${]}_{D}^{20}$ −10.5 (*c* 1.0, MeOH).

#### 3.2.10. Cell Culture and Treatments

The HDFs (5 × 105 cells) were seeded onto a 100Φ culture dish. HDFs were cultured in DMEM with 10% (*v*/*v*) feral bovine serum and 1% (*v*/*v*) penicillin/streptomycin at 37 °C and 5% CO_2_ for 48 h. Monolayer cultures of the HDFs in the culture dish were washed with PBS and cultured in serum free media for 24 h. HDFs were washed twice with PBS and exposed to UVB irradiation (5 mJ/cm^2^). After removal of the PBS, the HDFs were treated with each DHPV isoform in serum-free culture media for 24 h. The HDFs were harvested to extract RNA or protein.

#### 3.2.11. Quantitative Real-time Polymerase Chain Reaction

Total RNA was extracted using TRIzol reagent (Invitrogen, Carlsbad, CA, USA). The total RNA was quantified via nanodrop, and equal amounts (1.5 μg) of total RNA was reverse-transcribed using MMLV reverse transcriptase. To quantify the MMP-1 RNA expression revel, the cDNA was amplified with specific primers. The sequences of the PCR primers used for the amplifications of GAPDH and MMP1 were as follows. GAPDH forward: 5′-ACCACAGTCCATGCCATCAC-3′, GAPDH reverse: 5′-TCCACCACCCTGTTGCTGTA-3′; MMP1 forward: 5′-CTGCTTACGAATTTGCCGACAGA-3′, MMP-1 reverse: 5′-GTTGTAGGGAAGCCAAAGGAGCTG-3′. Quantitative real time PCR analysis was performed using the Power Green PCR Master Mix on a CFX Connect^TM^ Real-Time PCT Detection System (Bio-Rad, Hercules, CA, USA). GAPDH was used as the normalization gene in these studies. The relative expression levels of the target genes were given by 2^ΔΔCt^. GAPDH and MMP-1 were amplified for 40 cycles as follows: denaturation at 95 °C for 30 s, annealing at 60 °C for 30 s, and extension at 72 °C for 30 s for 40 cycles. The experiments were performed in triplicate.

#### 3.2.12. Western Blot Analysis

The HDFs were lysed with PRO-PREP^TM^ protein extraction solution (iNtRON Biotechnology, MA, USA). The cell lysates were separated by sodium dodecyl sulfate-polyacrylamide gel electrophoresis and transferred to 0.2 μm pore size PVDF membrane (Millipore, Burlington, MA, USA). The membrane was blocked with 3% BSA (RMBIO, Missoula, MT, USA) in Tris-buffered saline Tween-20 for 1 h and incubated with specific primary antibody at 4 °C overnight against MMP-1 (1:1000, Abcam, Cambridge, MA, USA), β-actin (1:3000, Abcam, Cambridge, MA, USA), followed by incubation with IgG-HRP (1:1000, GeneTex, Irvine, CA, USA). Protein bands were visualized by ClarityTM Western ECL Blotting Substrates (Bio-Rad, Hercules, CA, USA).

## 4. Conclusions

In conclusion, we developed a scalable four-step synthetic route (41% overall yield) for optically pure DHPVs. Our unique synthetic procedure, a key part of which involved efficient construction of the *γ*-valerolactone moiety via an oxidative lactonization, enabled us to elucidate the different biological activities of each metabolite. In particular, we confirmed that the metabolites of the flavan-3-ols possess different suppressive effects in the photo-induced MMP-1 protein expression, which are quite dependent on the C4-stereocenter of the DHPV.

Based on these results, further studies, including the structure-activity relationship and mode of action on DHPVs are in progress.

## Figures and Tables

**Table 1:**
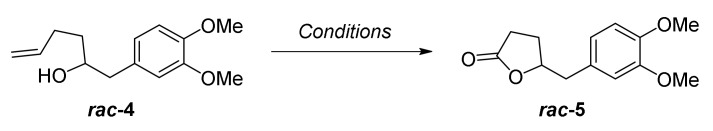
Optimization of oxidative lactonization conditions.

Entry	Condition (Equivalent)	Result
1	(i) OsO_4_ (0.02), NMO (4.0) in acetone/water (ii) NaIO_4_ (2.0) in THF/water (iii) NaOAc (2.0), PCC (2.0) in CH_2_Cl_2_	71% (3 steps)
2	(i) OsO_4_ (0.01), NaIO_4_ (4.0) in dioxane/water (ii) NaOAc (2.0), PCC (4.0) in CH_2_Cl_2_	51% (2 steps)
3	(i) OsO_4_ (0.01), NaIO_4_ (4.0), pyridine (2.0) in dioxane/water (ii) NaOAc (2.0), PCC (4.0) in CH_2_Cl_2_	64% (2 steps)
4	(i) OsO_4_ (0.01), NaIO_4_ (4.0), 2,6-lutidine (2.0) in dioxane/water (ii) NaOAc (2.0), PCC (2.0) in CH_2_Cl_2_	73% (2 steps)
5	OsO_4_(0.01), Oxone^®^ (4.0) in DMF	N.R. ^a^ (1 step)

*^a^* No reaction. The bold is lactonization condition after optimization.

## Supplementary Materials

The following are available online, Copies of NMR spectra (^1^H and ^13^C) and Chiral HPLC chromatogram of relevant compounds.
