# Supplementary material for: Concise Synthesis of Catechin Metabolites 5-(3′,4′-Dihydroxyphenyl)-γ-valerolactones (DHPV) in Optically Pure Form and Their Stereochemical Effects on Skin Wrinkle-Reducing Activities
Source: Molecules. 2020 Apr 23;25(8):1970. doi: 10.3390/molecules25081970 (PMC7221625; doi:10.3390/molecules25081970)
Supplement: Supplementary file 1 [file molecules-25-01970-s001.pdf]

Concise Synthesis of Catechin Metabolites 5-(3′,4′-Dihydroxyphenyl)-γ-Valerolactones (DHPV) in Optically Pure Form and Their Stereochemical Effects on Skin Wrinkle-Reducing Activities

Joonseong Hur[1], A-Ram Kim[2], Hyun Su Kim[3], Changjin Lim[4], Taewoo Kim[3], Tae-Aug Kim[2], Jaehoon Sim[5,*], Young-Ger Suh[3,*]

[1] Natural Products Research Institute, Korea Institute of Science and Technology (KIST), 679 Saimdang-ro, Gangneung 25451, Republic of Korea

[2] Department of Biochemistry, School of Medicine, CHA University, 335 Pangyo-ro, Bundang-gu, Seongnam 13496, Gyeonggi-do, Republic of Korea

[3] Department of Pharmacy, College of Pharmacy and Institute of Pharmaceutical Sciences, CHA University, 120 Haeryong-ro, Pocheon 11160, Gyeonggi-do, Republic of Korea

[4] College of Pharmacy, Jeonbuk National University, Jeonju 54896, Republic of Korea

[5] College of Pharmacy, Chungnam National University, Daejeon 34134, Republic of Korea

***** Correspondence: ygsuh@snu.ac.kr or ygsuh@cha.ac.kr; Tel.: +82-31-850-9300 (Y.-G.S.), jsim@cnu.ac.kr; Tel.:+82-42-821-5938 (J.S.)

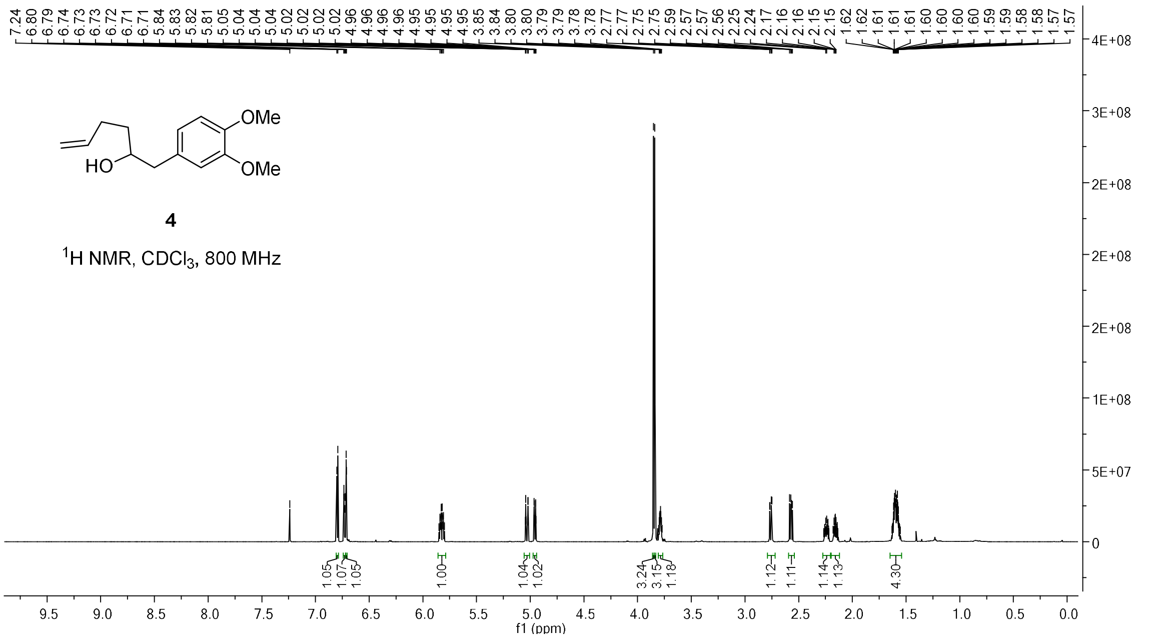

Fig. 1S ^{1}H NMR of **4**

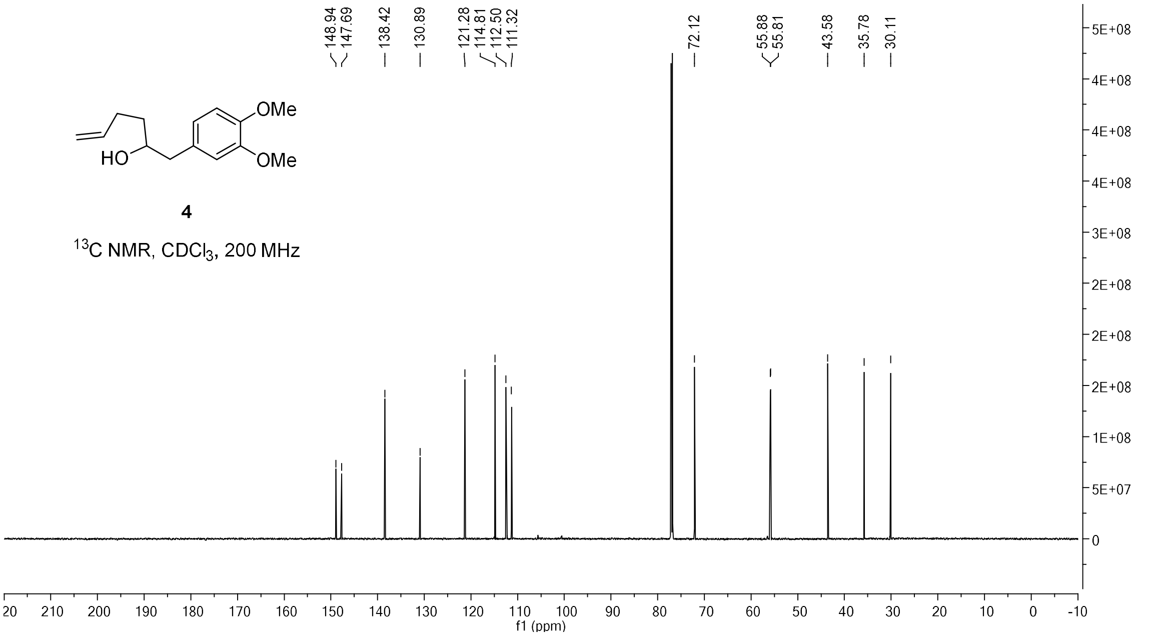

Fig. 2S ^{13}C NMR of **4**

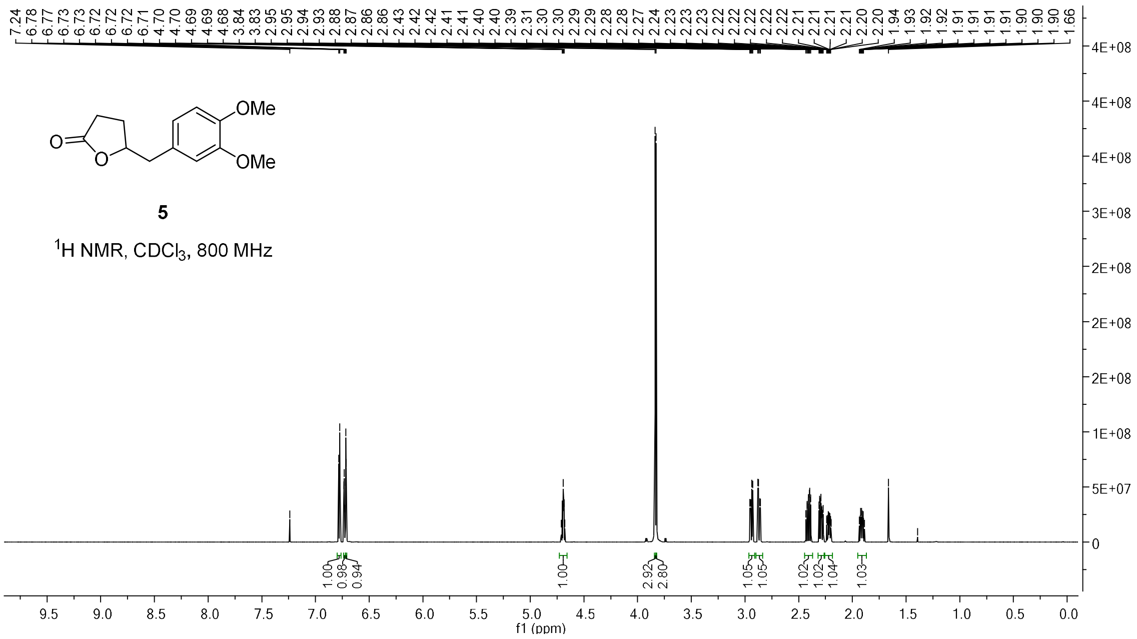

Fig. 3S ^{1}H NMR of **5**

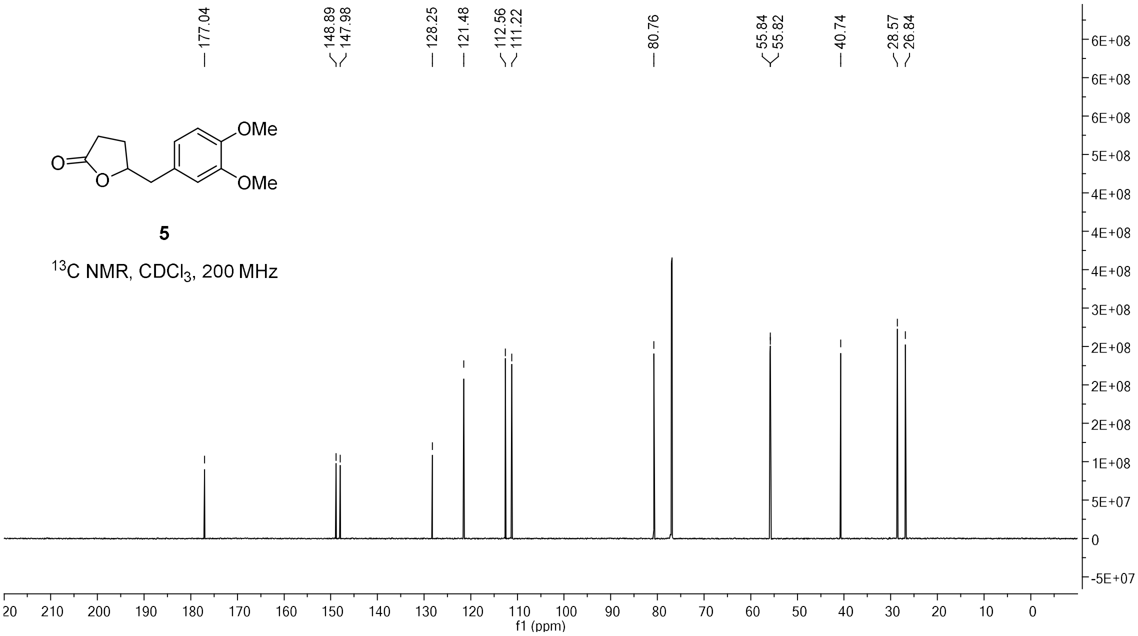

Fig. 4S ^{13}C NMR of **5**

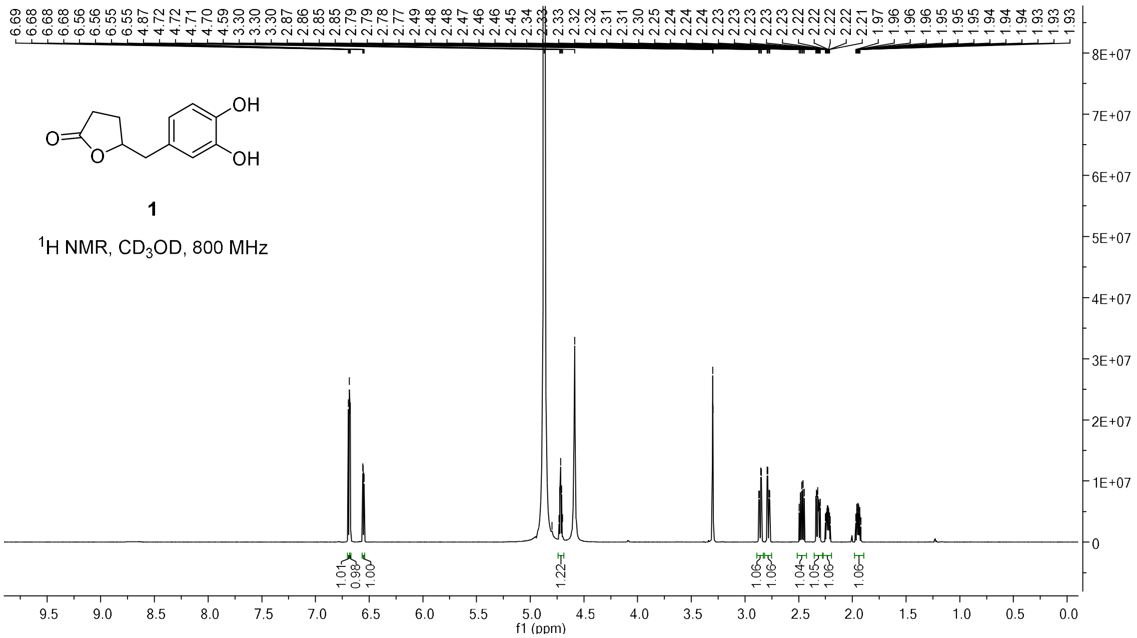

Fig. 5S ^{1}H NMR of **1**

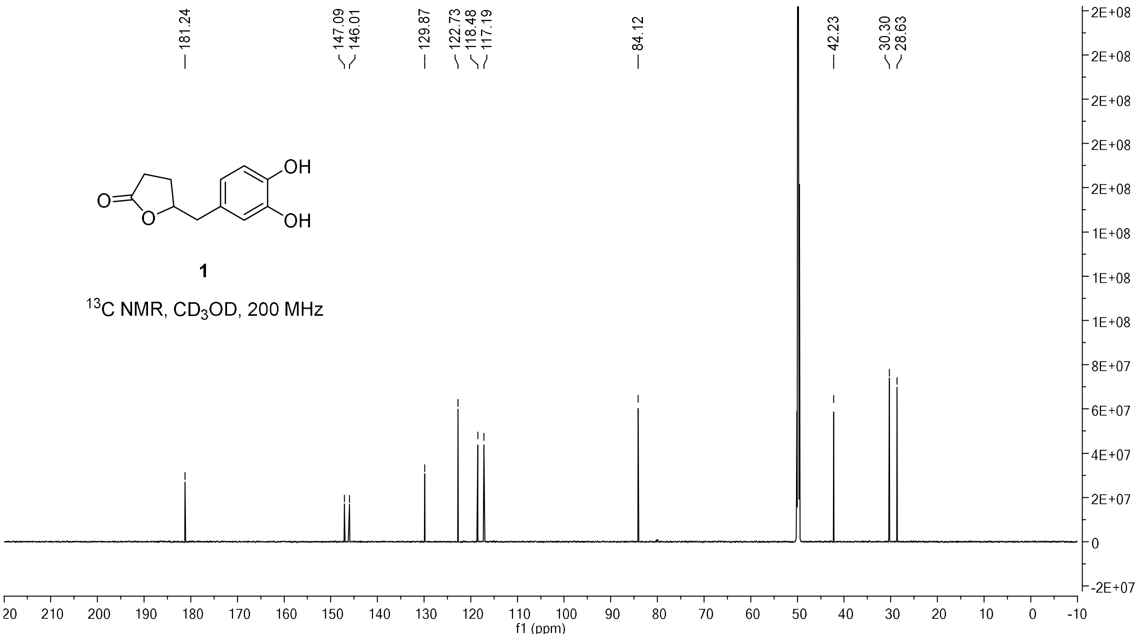

Fig. 6S ^{13}C NMR of **1**

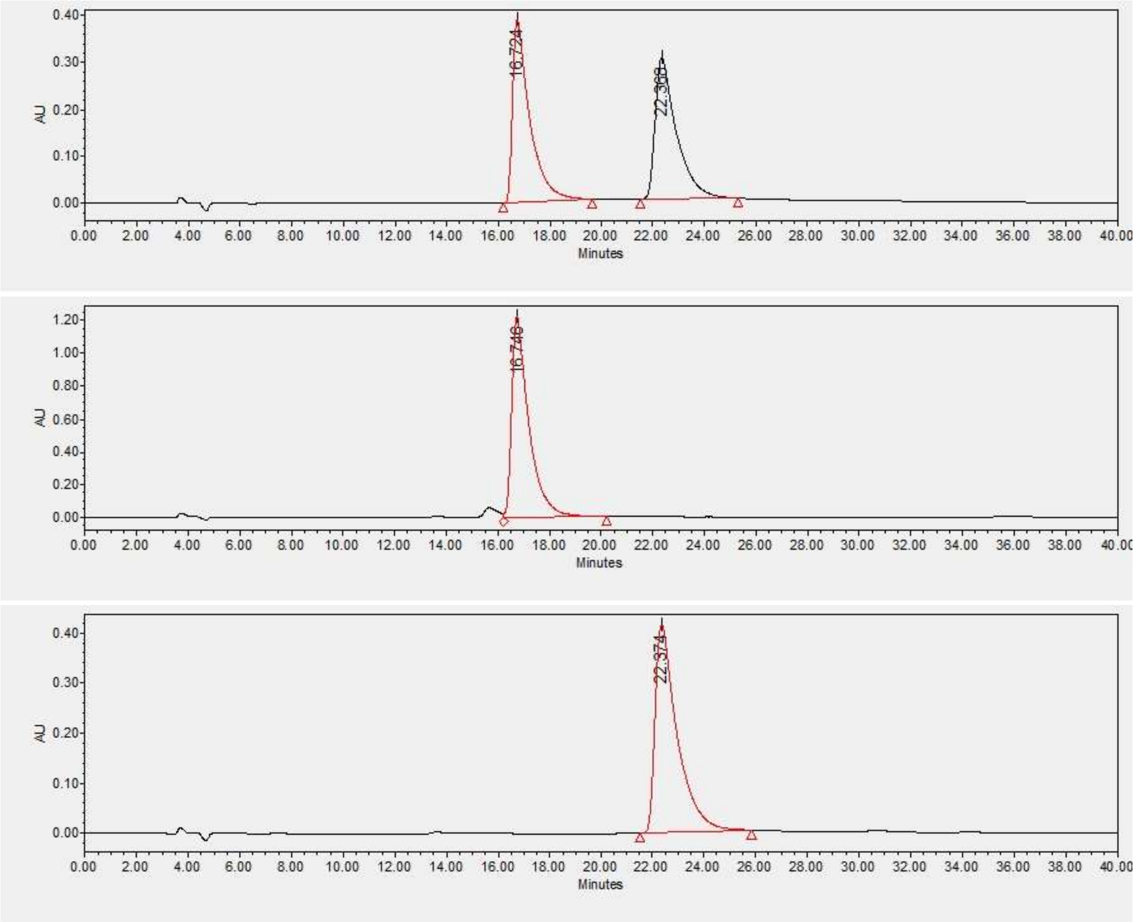

Fig. 7S Chiral HPLC chromatogram of *rac*-**1** (top), (*R*)-**1** (middle), (*S*)-**1** (bottom). All chromatograms were obtained under the following conditions; Column, DAICEL CHIRALPAK® AD-H, 4.6 x 250 mm, 5 µm; detection, UV 210 nm; flow rate 0.8 mL/min; Mobile phase, A: Isopropylalcohol, B: Hexane; Isocratic, A:B = 15:65
